# Foetal hepatic progenitor cells assume a cholangiocytic cell phenotype during two-dimensional pre-culture

**DOI:** 10.1038/srep28283

**Published:** 2016-06-23

**Authors:** Kazuya Anzai, Hiromi Chikada, Kota Tsuruya, Kinuyo Ida, Tatehiro Kagawa, Yutaka Inagaki, Tesuya Mine, Akihide Kamiya

**Affiliations:** 1 Department of Molecular Life Sciences, Tokai University School of Medicine, 143 Shimokasuya, Isehara, Kanagawa 259-1193, Japan; 2 Department of Gastroenterology, Tokai University School of Medicine, 143 Shimokasuya, Isehara, Kanagawa 259-1193, Japan; 3 Department of Regenerative medicine, Tokai University School of Medicine, 143 Shimokasuya, Isehara, Kanagawa 259-1193, Japan

## Abstract

Liver consists of parenchymal hepatocytes and other cells. Liver progenitor cell (LPC) is the origin of both hepatocytes and cholangiocytic cells. The analyses of mechanism regulating differentiation of LPCs into these functional cells are important for liver regenerative therapy using progenitor cells. LPCs in adult livers were found to form cysts with cholangiocytic characteristics in 3D culture. In contrast, foetal LPCs cannot form these cholangiocytic cysts in the same culture. Thus, the transition of foetal LPCs into cholangiocytic progenitor cells might occur during liver development. Primary CD45^−^Ter119^−^Dlk1^+^ LPCs derived from murine foetal livers formed *ALBUMIN (ALB)*^+^*CYTOKERATIN (CK)19*^−^ non-cholangiocytic cysts within 3D culture. In contrast, when foetal LPCs were pre-cultured on gelatine-coated dishes, they formed *ALB*^−^*CK19*^+^ cholangiocytic cysts. When hepatocyte growth factor or oncostatin M, which are inducers of hepatocytic differentiation, was added to pre-culture, LPCs did not form cholangiocytic cysts. These results suggest that the pre-culture on gelatine-coated dishes changed the characteristics of foetal LPCs into cholangiocytic cells. Furthermore, neonatal liver progenitor cells were able to form cholangiocytic cysts in 3D culture without pre-culture. It is therefore possible that the pre-culture of mid-foetal LPCs *in vitro* functioned as a substitute for the late-foetal maturation step *in vivo*.

The liver is the largest organ in the body and plays an important role in metabolism. Liver contains parenchymal cells and non-parenchymal cells. The parenchymal cells, also known as hepatocytes, deal with the main function of liver such as metabolism, detoxification, and synthesis of bile acid^1^,^2^. The non-parenchymal cells of the liver, such as cholangiocytes, stellate, sinusoidal endothelial, mesenchymal, and Kupffer cells, regulate the functions of mature hepatocytes through cell to cell interactions^3^. Among them, hepatocytes and cholangiocytes arise from same origin; they originate from the highly proliferative stem/progenitor cells in embryonic livers. The liver performs many complicated functions for maintaining homeostasis. Thus, we cannot substitute injured livers using artificial organs. Advance applications of regenerative medicine using stem/progenitor cells to create a liver *in vitro* and to transplant it into the patients, are in progress.

In mice, the foregut endoderm is stimulated by the soluble factors derived from the visceral endoderm and the septum transversum. Liver bud derived from the stimulated foregut migrates into the septum transversum and forms early liver organs^4^,^5^. In the liver bud, foetal LPCs, called hepatoblasts, expand and differentiate into mature liver cells, hepatocytes and cholangiocytes, during mid- to late-foetal liver development. In the first step of bile ductal development, foetal LPCs form single-layered condensed epithelial cells expressing biliary-specific proteins. These epithelial layers are known as the first ductal layer of ductal plates. Thereafter, the adjacent LPCs of the ductal plates differentiate into a biliary lineage cell, forming a second ductal plate layer. In the perinatal stage, these ductal layer cells give rise to the intrahepatic bile ducts. Several factors derived from the portal mesenchymal cells are important for these differentiation steps^6^,^7^. The concentration gradient of transforming growth factor beta (TGFβ) around the periportal region is important for the specification of foetal LPCs into cholangiocytic progenitor cells through the expression of cholangiocyte transcription genes, *Hepatocyte Nuclear Factor (Hnf) 1β* and *Hnf6*^8^. The *Jagged-1* gene is also important for bile duct formation and is related to the human genetic disease Alagille syndrome^9^,^10^. Foetal LPCs express *Notch* and deletion of the Notch ligand, Jagged-1, in portal mesenchymal cells causes malfunction of the ductal plate during perinatal liver development^11^. Thus, the induction of foetal LPCs into cholangiocytic cells by the cell-cell and extracellular soluble factors interaction is important for liver development.

Several markers, such as Dlk1, CD133, CD13, and EpCAM, are known to be expressed by foetal LPCs. For example, Dlk1-positive cells purified from murine embryonic day 13 (E13) foetal liver possess high proliferative ability and can differentiate into mature hepatocyte-like cells^12^. It has been recently described that Lgr5^+^ or EpCAM^+^ cells in the mature livers can form cholangiocytic cysts *in vitro* within the extracellular matrices in culture condition^13^,^14^. These cystic cells are able to expand over a long period with genetic stability. This suggests that the postnatal liver retains several cholangiocytic progenitor cells that are derived from foetal LPCs. In contrast, we found that the primary Dlk1^+^ progenitor cells derived from mid-foetal livers could not form cholangiocytic cysts in the same culture condition. Thus, some important changes that differentiate foetal LPCs into the cholangiocytic progenitor cells might occur during liver development. In this study, we revealed that pre-culture treatment on gelatine-coated dishes enabled the Dlk1^+^ foetal LPCs to become cholangiocytic progenitor cells, which could form cholangiocytic cysts *in vitro* culture. These cysts could expand over a period longer than 9 months *in vitro* and exhibited *ALB*-negative *CK19*-positive cholangiocyte characteristics. These results suggested that intrinsic changes in foetal LPCs are required for differentiation into cholangiocytic cells.

## Results

### Cholangiocytic cyst formation of progenitor cells derived from murine foetal livers

**Figure 1:**
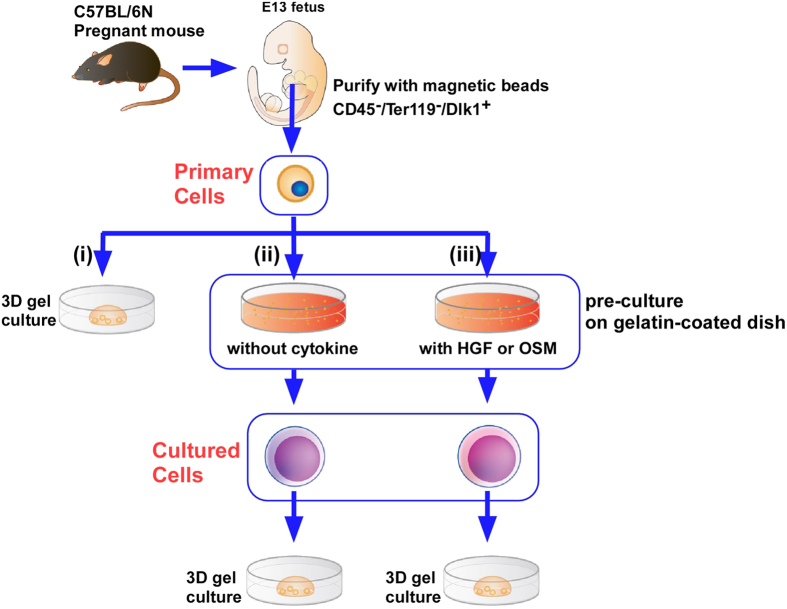
The schema of experiment protocol. CD45^−^Ter119^−^Dlk1^+^ foetal progenitor cells were purified using magnetic beads (‘primary cells’). (**i**) Primary cells were seeded directly into extracellular matrix gels. (**ii**) Primary cells were seeded to gelatine-coated plate and cultured for several days (2–9 days). Cells after pre-culture (‘cultured cells’) were seeded into gels. (**iii**) Primary cells were pre-cultured with either mock, OSM, or HGF for 7 days. These cells were seeded into gels. Illustrations were permitted for publication.

**Figure 2:**
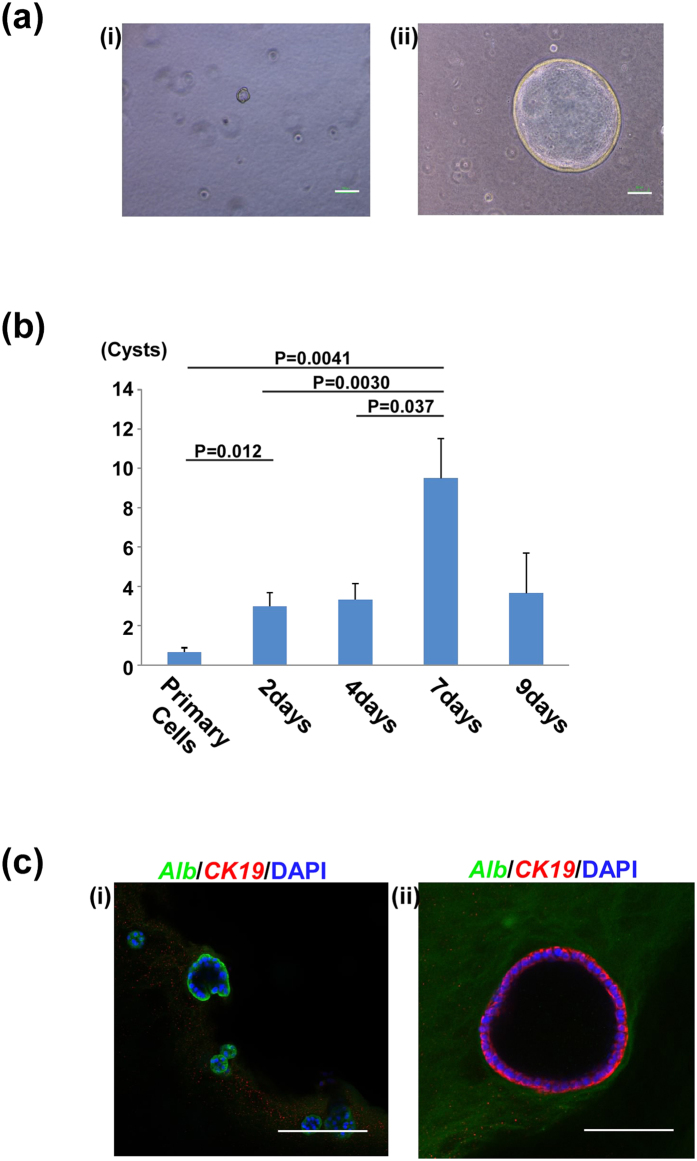
Cyst formation of murine liver progenitor cells. (**a**) Representative images of cysts after 12 days of gel culture. Scale bars, 100 μm. (i) Cyst derived from primary cells. (ii) Cyst derived from cultured cells. (**b**) The number of formed cysts affected the duration of 2D pre-culture. Primary cells were cultured for 2 to 7 days on gelatine-coated dishes. These cultured cells were seeded into matrix gels and the number of cysts was counted. Results are presented as mean ± SEM. Statistical analysis was performed with Student’s *t*-test (n  =  5). (**c**) Confocal microscopic images of cysts derived from foetal LPCs. Cysts were stained with anti-*ALBUMIN* (green) and anti-*CK19* (red). Nuclei were stained with DAPI (blue). (i) Cyst derived from primary cells exhibited *ALBUMIN*^+^*CK19*^−^ (ii) Cyst derived from cultured cells exhibited *ALBUMIN*^−^*CK19*^+^. Scale bars, 100 μm.

Hepatoblasts (foetal LPCs) can be purified as CD45^−^Ter119^−^Dlk1^+^ cells from E13 murine foetus^15^. Immediately after purification, cells (‘primary cells’) were seeded onto gelatine-coated dishes and cultured for several days. Either primary cells or the cells conditioned by the pre-culture (‘cultured cells’) were cultured in extracellular matrix gels (Fig. 1(i,ii)). After 12 days of 3D gel culture, primary cells formed a few, small cysts. On the other hand, cultured cells formed many large cysts (Fig. 2a). Furthermore, we varied the duration of the 2D pre-culture from 2 to 9 days, and counted the number of cysts with a diameter >50 μm (Fig. 2b). Cyst formation increased as the period of 2D pre-culture increased, and we concluded that 7 days of pre-culture was optimal for cholangiocytic cyst formation. Therefore, we used 7 days of pre-culture in the following experiments.

To confirm the characteristics of these cysts, they were stained with specific antibodies against *ALBUMIN (ALB)* and *CYTOKERATIN (CK)19*. Cysts derived from the primary cells expressed hepatocyte marker *ALBUMIN*, but did not express the cholangiocyte marker *CK19* (Fig. 2c(i)). In contrast, cysts derived from the cultured cells exhibited *ALB*-negative and *CK19*-positive, cholangiocytic characteristics (Fig. 2c(ii)). These data showed that primary foetal LPCs derived from E13 livers could not directly differentiate into cholangiocytic cysts in 3D culture conditions. They require 2D pre-culture to acquire the ability for cholangiocytic differentiation.

Next, we determined the characteristics of the primary cells and the pre-cultured cells. E13 LPCs were 2D pre-cultured for 1 day, 3 days, and 5 days and stained with specific antibodies against *CK19* and *Ki-67*. Most cells inoculated on culture plates were proliferating, as shown by expression of *Ki-67* (Supplementary Fig. S1). Primary cells without pre-culture (day 0) barely expressed the cholangiocytic marker *CK19*. Expression of *CK19* was induced during 2D pre-culture (day1, 3, and 5). In addition, the number of cells increased to almost 10 times during 2D pre-culture (Supplementary Fig. S2). These results suggest that primary cells begin to differentiate into the cholangiocytic lineage shortly after seeding onto gelatine-coated plates. Furthermore, they demonstrate a proliferative capacity throughout the pre-culture.

### Characterisation of cholangiocytic cysts derived from foetal LPCs

**Figure 3:**
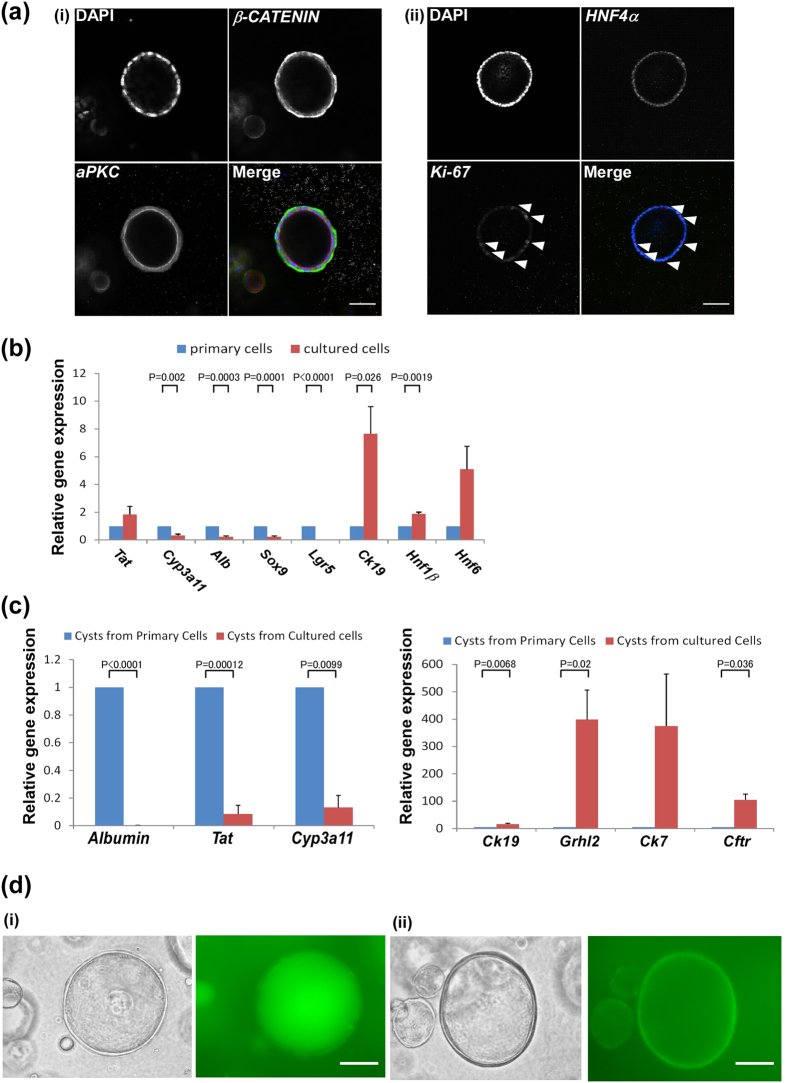
Characteristics of cysts from cultured foetal LPCs. (**a**) Confocal microscopic images of cysts derived from cultured cells. (i) *β-CATENIN* located at the basolateral region and apical protein kinase C (*aPKC*) located at the luminal region. (ii) *HNF4*α and Ki-67 were stained. Scale bars, 50 μm. Arrowheads indicate *Ki-67*-positive cells. Scale bars, 100 μm. (**b**) Gene expression of primary cells and cultured cells were analysed using quantitative RT-PCR. *Sox9* and *Lgr5* (progenitor markers), *Ck19*, *Hnf1β*, and *Hnf6* (cholangiocyte markers), and *Tat*, *Cyp3a11*, and *Albumin* (hepatocyte markers) were analysed. Expression level of primary cells was set to 1.0. Results are presented as mean ± SEM. Statistical analysis was performed with Student’s *t*-test (n  =  5). (**c**) Gene expression of cysts derived from primary and cultured cells were analysed using quantitative RT-PCR. *Tat*, *Cyp3a11*, and *Albumin* (hepatocytic markers) and *Ck19*, *Hnf1b*, *Hnf6*, and *Cftr* (cholangiocytic markers) were analysed. Expression level of primary cells was set to 1.0. Results are presented as mean ± SEM. Statistical analysis was performed with Student’s *t*-test (n  =  5). (**d**) Function of cysts derived from cultured cells *in vitro*. (i) Representative images showing accumulation of rhodamine 123 dye (green) to luminal space indicate functional transport of dye. (ii) Cysts incubated with verapamil before the addition of rhodamine 123. The activities of MDR1 were suppressed. Scale bars, 100μm.

Next, we analysed characteristics of cholangiocytic cysts derived from the foetal LPCs. We stained the cysts with specific antibodies such as *β-CATENIN*, *apical protein kinase C (aPKC)*, *hepatocyte nuclear factor (HNF) 4α* and *Ki-67*. *β-CATENIN* and *aPKC* were located in the basolateral and luminal regions, respectively (Fig. 3a(i)). In addition, the cysts were positive for hepatocyte transcription factor *HNF4*α and included *Ki-67* positive cells (Fig. 3a(ii)). Thus, cysts derived from the cultured cells had a high proliferative ability with cholangiocytic characters such as epithelial polarisation of cell surface proteins. However, they have an immature phenotype as shown by *HNF4*α expression.

We also analysed the expression of hepatocyte and cholangiocyte related-genes by quantitative PCR. Expression of cholangiocyte markers *Ck19* and *Hnf1β* was induced in the pre-culture condition. In contrast, expression of hepatocyte markers such as *Albumin* and *Cytochrome P450* (*Cyp3a11*) were down-regulated. In addition, progenitor markers such as *Sry* (*Sex Determining Region Y*)*-box 9* (*Sox9*) and *Leucine-rich-repeat-containing G-protein-coupled Receptor 5* (*Lgr5*) were down-regulated (Fig. 3b). Furthermore, compared to primary cells, cysts derived from cultured cells expressed extremely low levels of *Albumin*, *Tat*, and *Cyp3a11* (Fig. 3c, left panel). On the other hand, expression of cholangiocytic genes such as *Ck19*, *grainyhead like-2* (*Grhl2*), and *cystic fibrosis transmembrane conductance regulator* (*Cftr*) were induced (Fig. 3c, right panel) in formed cysts. These results suggested that 2D pre-cultures allow primary cells to obtain the ability of differentiating into cholangiocytic cells and that the extracellular matrix gel culture condition induced their ductal aspects and suppressed the hepatocytic phenotypes.

To assess the functional aspects of the cysts, we evaluated their ability to efflux rhodamine 123. Rhodamine 123, a fluorescent dye that is accumulated in the mitochondria, is an MDR1 substrate and a well-established tool for studying MDR1 transport activity. Inhibitors of MDR1-dependent transport, such as verapamil, have been found to decrease rhodamine 123 efflux from MDR1-expressing cells^16^. When rhodamine 123 was added to the 3D culture media, cysts derived from the cultured cells transported and showed accumulation of the dye into the luminal space, indicating normal transporter activity (Fig. 3d(i)). In the presence of 100 μM verapamil, rhodamine 123 was not transported to the luminal space (Fig. 3d(ii)). We counted the number of cysts that had accumulated rhodamine 123 into their luminal spaces. For both culture conditions, with or without verapamil, 15 images were chosen at random and the number of cysts with or without dye accumulation was counted. All cysts (n = 34) in the verapamil-negative culture had accumulated rhodamine 123, whereas all cysts (n = 30) in verapamil-positive culture had not. These results confirmed that movement of the dye indicated the specific activity of MDR1.

**Figure 4:**
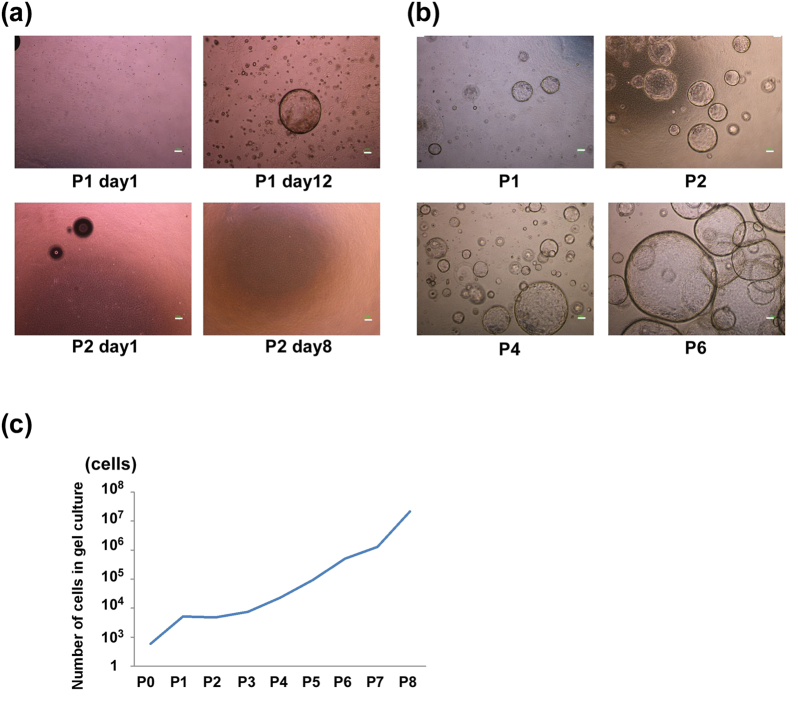
Proliferative ability of cysts derived from cultured cells. (**a**) Representative images of cysts derived from primary cells at each passage (P1, P2, P  =  passage) and 8 or 12 days of culture after passage. (**b**) Representative images of cysts derived from cultured cells at each passage (P1, P2, P4, P6). (**a,b**) Scale bars, 100 μm. (**c**) Growth curve of the cystic cells derived from pre-cultured LPCs for 7 days. The number of cells was counted at each passage.

We also observed long-term proliferative ability of cholangiocytic cysts in the *in vitro* gel culture. Primary and cultured cells were seeded into matrix gels and the cysts formed were passaged and dissociated using trypsin treatment. The number of isolated cells was counted at each passage step. Cysts derived from primary cells did not expand, whereas cysts derived from cultured cells maintained a stable growth ability (Fig. 4a,b). The number of cells in this cystic culture increased over a 9 month period *in vitro* (Fig. 4c). Collectively, foetal LPCs change their characteristics to cholangiocytic progenitor cells that exhibit continuous growth ability and the capacity to form cholangiocytic structures with normal transporter ability through 2D pre-culture *in vitro*.

### Hepatic maturation factors inhibited acquisition of the ability of LPCs to form cholangiocytic cysts

**Figure 5:**
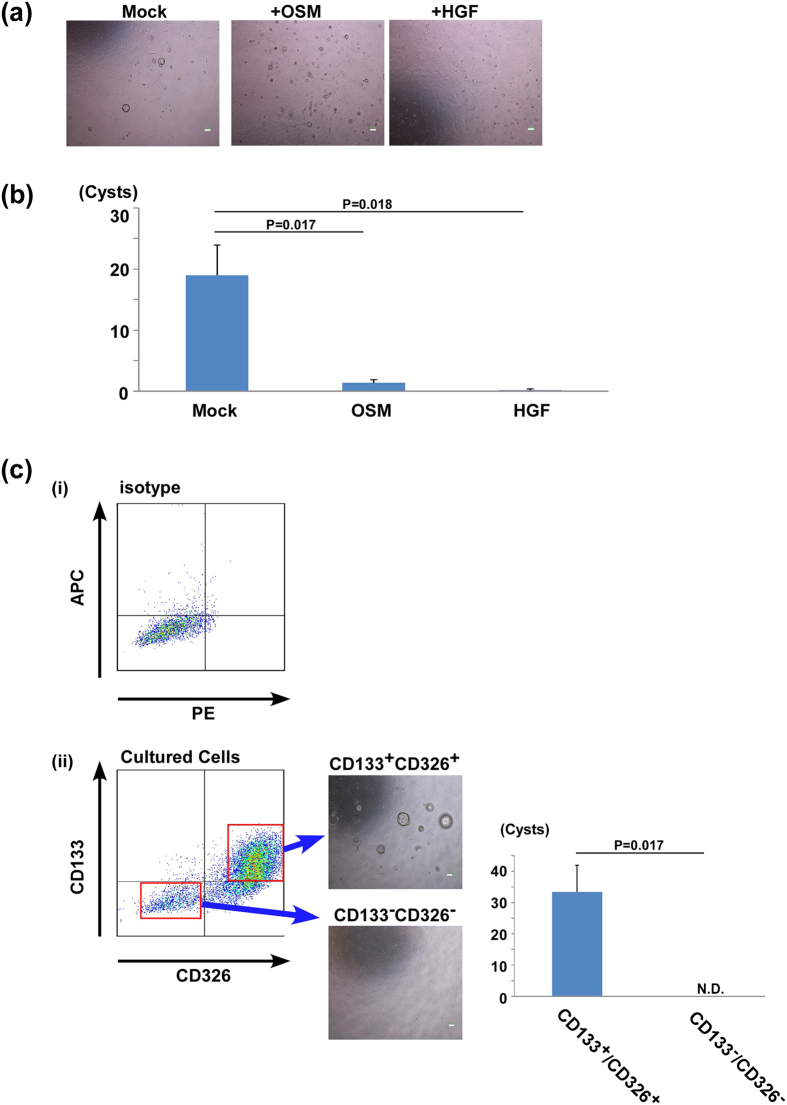
The influence of cytokine stimulation during pre-culture condition on cholangiocytic differentiation. (**a**) Representative images of cysts derived from cultured cells with or without cytokines during 2D pre-culture. Scale bars, 100 μm. (**b**) The number of cysts after *in vitro* gel culture. The results are presented as the mean ± SEM. Statistical analysis were performed with Student’s *t*-test (n  =  5) (**c**) Flow cytometry analyses of cultured cells without cytokines. (i) Isotype control, (ii) Expression of CD326 and CD133. CD133^+^CD326^+^ cells and CD133^−^CD326^−^ cells were sorted and seeded into extracellular matrix gels. CD133^+^CD326^+^ cells but not CD133^−^CD326^−^ cells could form cysts *in vitro* (N.D, not detected). The results are presented as the mean ± SEM. Statistical analysis were performed with Student’s *t*-test (n  =  5). Scale bars, 100 μm. Mock, cells were pre-cultured without cytokines.

Hepatocyte growth factor (HGF) is a cytokine that is involved in the development of liver in the foetus and in the cell division of hepatocytes in adults. On the other hand, oncostatin M (OSM) is a member of the interleukin-6 (IL-6) family cytokines secreted from hematopoietic cells in mid-foetal livers that promotes hepatic maturation^17^,^18^,^19^. To examine the effect of these hepatocytic factors on pre-culture, primary cells were pre-cultured for 7 days in the absence or presence of HGF or OSM (Fig. 1(iii)). The pre-culture with either HGF or OSM significantly suppressed the formation of cholangiocytic cysts in the sequential gel culture (Fig. 5a,b), suggesting that the stimulus of OSM and HGF suppresses the differentiation of foetal LPCs to cholangiocytic progenitor cells.

Next, we examined the expressional change of cell surface antigens using flow cytometry analysis. LPCs pre-cultured with or without the soluble factors were stained with an APC-conjugated anti-CD133 antibody and PE-conjugated antibody against several cell surface molecules (shown in Supplementary Table S1). CD133, also known as Prominin-1, is a common cell surface marker in foetal and adult bi-potent progenitor cells^20^,^21^. As shown in Supplementary Fig. 3S, expression of almost all of the cell surface antigens barely changed by the addition of HGF or OSM. In contrast, the ratio of CD133 positive cells apparently decreased under stimulation with HGF or OSM, suggesting that hepatic maturation induced by HGF and OSM suppressed the expression of CD133 on LPCs. In addition, flow cytometry analysis showed that CD133 was co-expressed with CD326, an epithelial cell marker. LPCs pre-cultured for 7 days were divided into CD133^+^CD326^+^ cells and CD133^−^CD326^−^ cells. Thus, both cells were cultured *in vitro* in extracellular matrix gels. CD133^+^CD326^+^ cells, but not CD133^−^CD326^−^ cells, formed cholangiocytic cysts effectively (Fig. 5c). During the 2D pre-culture, foetal LPCs began transitioning into cholangiocytic progenitor cells, and the changes were suppressed by the stimuli from HGF and OSM. Besides, CD133 and CD326 were specific markers for cholangiocytic progenitor cells.

### Cyst formation was reduced by JNK and PI3K/Akt signal inhibition

**Figure 6:**
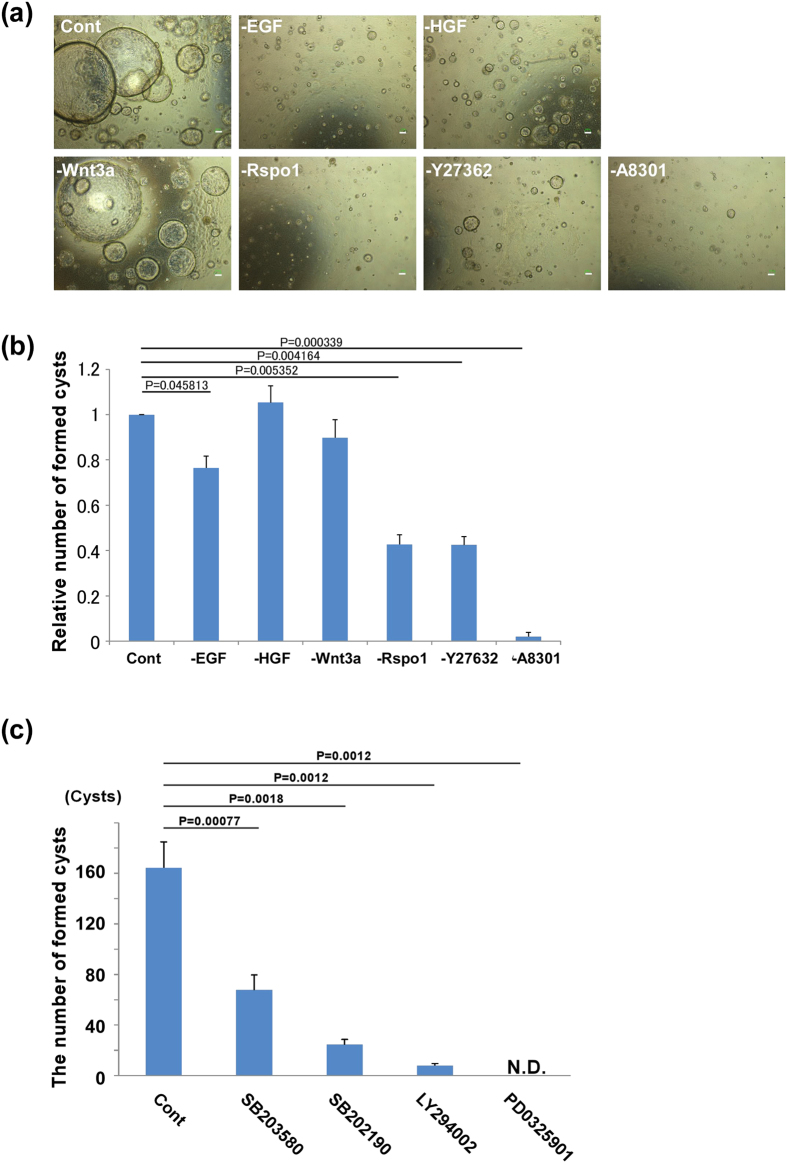
Requirements of cell signalling molecules for cholangiocytic cyst formation. (**a,b**) Cysts cultured in the absence of suitable cytokines and reagents. ‘-EGF media’ means EGF was excluded but the other 5 reagents (HGF, Wnt3a, R-spondin-1, Y27632, and A8301) were included. EGF, R-spondin-1, and A83-01 were strictly required for the formation of cholangiocytic cysts. (**a**) Representative images of cysts. Scale bars, 100 μm. (**b**) For quantitation, cells were seeded into each 3D gel and cultured for 12 days in suitable culture media. The number of cells seeded in 3D gels was slightly different between sets, therefore we set the control as 1.0 and the relative number was calculated. Results are presented as mean ± SEM. Statistical analysis was performed with Student’s *t*-test (n = 9). (**c**) The number of cysts after 3D gel culture with various inhibitors. During 3D gel culture, p38 signal inhibitor (SB203580, SB202190), PI3K inhibitor (LY294002), and MAPK inhibitor (PD325901) were added. The addition of inhibitors started from 3 day of culture. The results are presented as the mean ± SEM. Statistical analysis were performed with Student’s *t*-test (n = 5).

In our study, the cyst formation media contained 4 soluble factors and 2 signal inhibitors such as EGF, HGF, Wnt3a, R-spondin-1, ROCK inhibitor Y-27632, and TGF-β inhibitor A-8301. To evaluate the individual effect of these cytokines and inhibitors precisely, we coordinated one-reagent extracted media. For example, ‘-EGF media’ did not include EGF but included other 5 reagents. The cultured cells were cultured in 3D matrix gels with these media. The gel culture without A-8301 could barely form cholangiocytic cysts *in vitro*. In addition, removal of EGF, R-spondin-1 or Y-27632 from the culture significantly decreased the number of cysts that formed (Fig. 6a,b). Thus, we analysed expression of receptors for related factors such as EGF and R-spondin 1 in formed cysts. The expression of ErbB receptor family (the receptors of EGF) was expressed on formed cysts, especially about ErbB1 and ErbB2. Moreover, the cysts derived from the cultured cells relatively maintained the expression of Lgr family (the receptors of R-spondin 1) (Supplementary Fig. S4).

We examined the cell signalling pathways involved in cyst formation. Cultured cells were seeded into 3D matrix gels and cultured with SB203580 and SB 202190 (p38 signal inhibitor), AS601245 and SP600125 (JNK signal inhibitor), cyclopamine (Hedgehog signal inhibitor), PS48 (PDK1 signal inhibitor), LY293002 (PI3K/Akt signal inhibitor), DAPT (Notch signal inhibitor), and PD0325901 (MAPK signal inhibitor). The inhibition of p38, PI3K/Akt, and MAPK pathways significantly influenced cyst formation (Fig. 6c). These findings suggest that cholangiocytic differentiation of pre-cultured LPCs require appropriate signal transduction of p38, PI3K/Akt, and MAPK pathways, which are stimulated by growth factors such as EGF and R-spondin-1.

### Changes in hepatoblast characteristics during perinatal liver development

**Figure 7:**
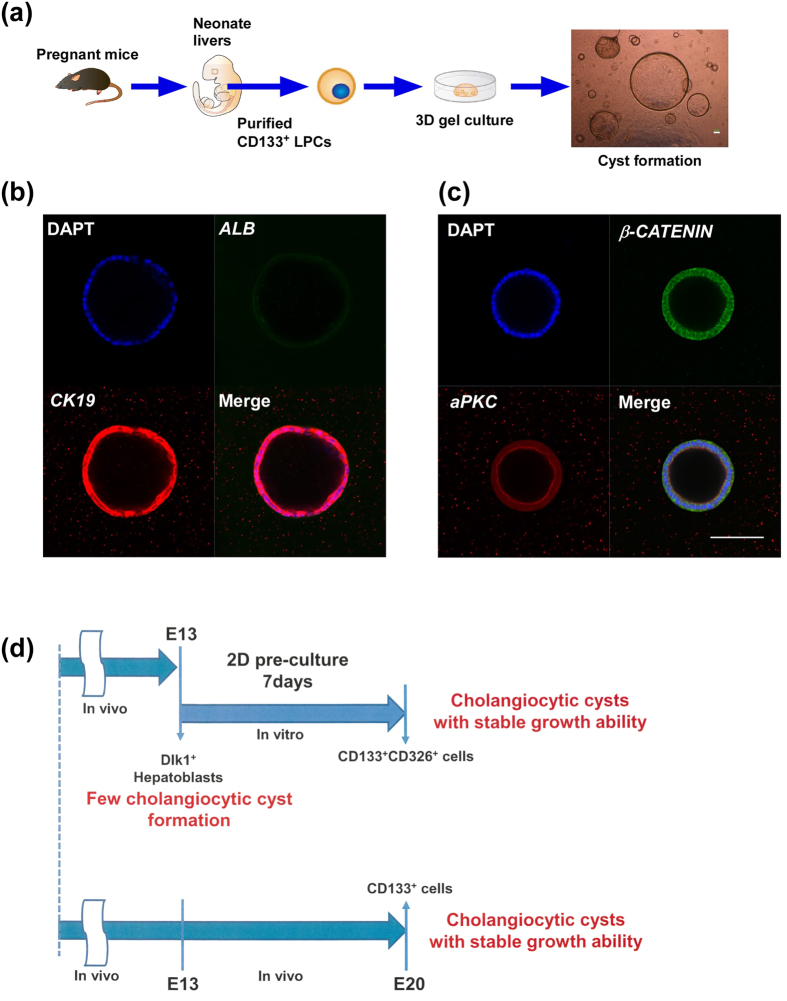
Neonatal CD133^+^ liver progenitor cells can form cysts without pre-culture condition. (**a**) Schema of experiment protocol. Neonatal liver were minced and purified with CD133 antibodies, and directly seeded into 3D extracellular matrix gels. After 7 days of culture, they formed many cysts. (**b**) Confocal microscopic images of cysts derived from neonatal CD133^+^ liver cells. (i) Cysts derived from neonatal CD133^+^ cells expressed cholangiocytic marker *CK19* but not hepatocytic marker *ALBUMIN*. (**c**) The cysts had epithelial polarity that *β-CATENIN* (green) located basolateral region and *aPKC* (red) located luminal region. Scale bars, 100 μm. (**d**) Schema of foetal LPC differentiation into cholangiocytic progenitor cells *in vitro* and *in vivo*. Illustrations were permitted for publication.

As shown above, we demonstrated that E13 LPCs require 7 days pre-culture to obtain the ability to form cholangiocytic cysts. Thus, we speculated that several maturation times are necessary for the acquisition of a cholangiocytic phenotype, both *in vitro* and *in vivo*, and attempted to examine liver progenitor cells in neonates, which correspond to E20. Because neonatal liver cells do not express Dlk1, we employed CD133 as a purification marker for the neonatal liver progenitor cells. CD133^+^ neonatal liver cells were purified with magnetic beads, and directly seeded into 3D matrix gels. As expected, neonatal CD133^+^ LPCs could easily form cysts without 2D pre-culture (Fig. 7a), and these cysts showed stable proliferative ability and could expand *in vitro*. In addition, immunohistochemistry revealed that the cysts exhibited *ALBUMIN*^−^*CK19*^+^ cholangiocyte characters and basolateral *β-CATENIN* and luminal *aPKC* localization (Fig. 7b,c). In addition, HNF4α and Ki67 expression was also detected (Supplementary Fig. S5). These findings suggest that CD133 is a useful marker for purifying cholangiocytic progenitor cells and neonatal liver cells.

Next, we performed a bi-potent colony forming assay to analyze the characteristics of CD133^+^ neonatal liver cells^22^. CD133^+^ E13 primary LPCs, CD133^+^ cells derived from 2D pre-culture of E13 LPCs, and CD133^+^ neonatal liver cells were sorted and seeded at a low density onto MEF feeder cells. These cells had a high proliferative capacity, as indicated by large colony formation from single cells (Supplementary Fig. S6). Colonies derived from individual cells were stained with antibodies against *ALBUMIN* and *CK19*. The CD133^+^ E13 liver primary cells formed *ALB*^+^/*CK19*^−^ colonies, whereas CD133^+^ cells derived from 2D pre-culture of E13 LPCs and CD133^+^ neonatal liver cells formed mainly *ALB*^−^/*CK19*^+^ cholangiocytic colonies. These results suggest that the CD133^+^ cells derived from 2D pre-culture of E13 LPCs and CD133^+^ neonatal liver cells have similar characteristics to cholangiocytic progenitor cells (Fig. 7d).

## Discussion

Foetal LPCs in the liver have a high proliferative ability and bi-potency to differentiate into hepatocytes or cholangiocytes. In the late-foetal and perinatal liver, some foetal LPCs near the portal veins commit to the cholangiocytic lineage and form mature bile ductal structures. Several extracellular signalling pathways are known to be important for these steps. However, the intrinsic changes that regulate the differentiation of foetal LPCs into cholangiocytic progenitor cells remain largely unknown. In this study, we demonstrated that cholangiocytic progenitor cells could be induced from foetal LPCs by culturing them on gelatine-coated dishes for 7days. The generations of bile duct start from the activation of Notch signal pathway by intimate interaction of hepatoblasts and fibroblasts, which exhibit Jagged-1 near the portal vein^23^. The stimulation of TGFβ that is secreted from vascular endothelial cells and fibroblasts promote maturation of hepatoblasts toward cholangiocytes^8^. These cytokines contribute to the formation of the cholangiocytic monolayer called the ‘ductal plate^6^. Thus, it is possible that these extracellular signals contribute to the intrinsic changes in foetal LPCs for cholangiocytic differentiation. We found that primary LPCs derived from E13 foetal liver could not differentiate into cholangiocytic structures *in vitro*. In contrast, primary CD133^+^ progenitor cells derived from neonatal liver were able to form cholangiocytic structures. Using an *in vitro* colony formation assay, we found that both 2D-pre cultured E13 LPCs and neonatal liver-derived progenitor cells, but not primary E13 LPCs, formed many *ALB*^*−*^*CK19*^*+*^ cholangiocytic colonies. These results suggest that the 7 days of 2D pre-culture induced a switch from foetal LPCs to cholangiocytic progenitor cells, which are similar to progenitor cells derived from neonatal liver. In addition, our culture system could be useful for analysis of the intrinsic changes of foetal LPCs towards cholangiocytic differentiation.

In pre-culture conditions without HGF and OSM, CD133^+^CD326^+^ cells were maintained and were able to form cholangiocytic cysts in 3D matrix gel culture. In contrast, CD133 expression was significantly suppressed by addition of HGF or OSM to the pre-culture conditions. Therefore, expression of CD133 could be correlative with a cholangiocytic progenitor ability. HGF and OSM are known as hepatocytic maturation factors for foetal LPCs^19^,^24^, and they induce the expression of several liver maturation markers in E13 foetal liver culture. Thus, when these factors were added to the 2D pre-culture step, they inhibited the cholangiocytic differentiation of foetal LPCs and permitted the progression of hepatocytic differentiation. Elucidating the effects of HGF and OSM will permit further understanding of the processes that control the fate of hepatoblasts.

In 3D culture conditions, EGF, R-spondin1 and the TGFβ signal inhibitor, A-8301, were required for cholangiocytic cyst formation. Lgr5 is a stem/progenitor cell marker for intestinal, gastric, and adult liver^25^. R-spondin-1, the Lgr family ligand, is also known to be important in the formation of organoid, derived from several tissues such as intestine, colon, and pancreas^26^,^27^. Thus, the Wnt signal regulation controlled by Lgr receptor family is an important factor for the formation of cystic structures. For *in vitro* expansion and differentiation of stem/progenitor cells, suitable extracellular environment that stimulated the cell signalling molecules was required. In this study, we revealed that the activation of ERK, p38, MAPK, and PI3K pathways were important for the formation of cholangiocytic cysts *in vitro*, suggesting that these signalling molecules are important for *in vivo* bile ductal formation. Ductal plates in the periportal region, derived from foetal LPCs, mature into the bile ductal network during prenatal and postnatal development. Periportal fibroblasts expressed several extracellular factors and were important for the maturation of bile ductal cells. For example, TGFβ derived from portal mesenchymal cells is important in the first step of ductal formation. In contrast, in this study, we found that the inhibition of TGFβ receptor signal using A-8301 was critical for the expansion step of cholangiocytic cells in cyst formation. The analyses of these molecular mechanisms might be useful for the induction of functional cholangiocytic cells *in vitro*.

There are several bile duct-related diseases such as Alagille syndrome, polycystic liver disorder, and malignant tumours (i.e. intrahepatic cholangiocarcinoma), and suitable disease models are required to evaluate the therapeutic and side effects of new medicines. In this study, we revealed that 2D pre-culture of E13 LPCs is required for cholangiocytic differentiation *in vitro*. During the pre-culture, the expression of several cholangiocytic-related genes such as *Ck19*, *Hnf1β*, and *Hnf6* was induced. However, the transcriptional mechanisms regulating these genes remain unknown. It has previously been reported that several transcription factors, such as *Sall4* and *Grhl2,* are involved in the cell fate decision of foetal LPCs^28^,^29^. Future analyses to identify the key factors that regulate the switch from foetal liver LPCs into cholangiocytic progenitor cells using our culture system will improve cholangiocytic differentiation efficiency *in vitro* and support the establishment of *in vitro* bile ductal disease models.

## Methods

### Animals

C57BL/6N mice were purchased from Nihon SLC (Shizuoka, Japan). All animal experiments were performed in accordance with the approved guidelines and these protocols were approved by the Institutional Animal Care and Use Committee in Tokai University (permit number: 144008).

### Isolation of CD45^−^Ter119^−^Dlk1^+^ hepatoblasts from foetal liver

Isolation of CD45^−^Ter119^−^Dlk1^+^ cells was described previously^30^. Briefly, minced embryonic liver tissues from E13 mice were dissociated with 0.05% collagenase Type 1 (Worthington, Lakewood, NJ, USA). Contaminated hematopoietic cells were excluded by Dyna Mag^TM^ 15 (Life Technologies, Carlsbad, CA, USA) with bioitin-conjugated-anti-CD45 and anti-Ter119 antibodies, and Dynabeads^®^ MyOne^TM^ streptavidin C1 (Life Technologies). Subsequently, Dlk1-positive cells were selected and purified by MACS technology (Miltenyi Biotec, Bergisch Gladbach, Germany) with anti-Dlk1 antibody (Preadipocyte factor-1, Medical and Biological Laboratories, Nagoya, Japan). These purified CD45^−^Ter119^−^Dlk1^+^ cells have the character of murine foetal progenitor cells and we call them ‘primary cells’ in our study.

### Two-dimensional (2D) pre-culture of foetal hepatoblasts

About 2 × 10^5^ primary cells were seeded per well on 0.1% gelatine-coated 12-well plates for 2 to 9 days in hepatocyte culture media: Dulbecco’s modified Eagle’s Medium (DMEM, Sigma, St Louis, MO, USA) supplemented with 10% foetal bovine serum (FBS), 1 × MEM Non-Essential Amino Acids Solution (Life Technologies), insulin-transferrin-selenium (Life Technologies), 10^−7 ^M dexamethasone (Sigma), and penicillin-streptomycin-glutamate (Life Technologies). Culture media were changed every other day. In some experiments, we added cytokine, OSM (R&D systems, Minneapolis, MN, USA) or HGF (Peprotech, Rocky, NJ, USA) during 2D pre-culture. In our study, we call the cells subjected to 2D pre-culture as ‘cultured cells’.

### Three-dimensional (3D) culture of cells

After 2D pre-culture, cells were detached by 0.5% trypsin-EGTA solution (Sigma) from the plate and washed with DMEM and 10% FBS. The cell pellet (5000 cells per a well of 24-well culture plate) was mixed with extracellular matrices that consisted of Matrigel (BD Bioscience, San Jose, CA, USA) and Collagen Type-1 (Nitta Gelatin, Japan). In Fig. 2 (b), 2500 cells per well of 24-well culture plates were used. After 10 min incubation, gels become solid and the culture medium was added. The culture medium was a 1∶1 mixture of H-CFU-C medium and DMEM/F-12 supplemented with 2% B27 supplement, 0.25 μmol A-83-01 (Wako Pure Chem., Osaka, Japan), 10 μmol Y-27632 (Wako Pure Chem.), 20 ng/ml EGF (Peprotech), 40 ng/ml HGF, 40 ng/ml recombinant human Wnt-3a (R&D Systems), and 100 ng/ml recombinant human R-spondin 1 (Peprotech). Media was changed at every 3 days. At 8 and 12 days, the number of cysts with >50 μm diameter was counted.

### Cyst passage and cell number counting

For passage, culture media were removed and gels were washed with PBS. Cell Recovery Solution (Corning) were added and gels were mechanically dissociated into small fragments. After shaking for 30 min on ice, cells were isolated using trypsin and the number of cells was counted by trypan blue exclusion at the indicated time points. 2,500 or 5,000 cells were mixed with 3D culture matrices gels and seeded into 24-well plate again. Passage was performed in a 1:4–1:8 split ratio, once every 10–15 days.

### Immunohistochemistry

The 3D gels were washed with PBS(+) and collagens were mildly digested with collagenase 1 (Sigma) for about 10 min at 37 °C. After washing with PBS(+), cyst cells were fixed with 4% paraformaldehyde for 30 min. After washing 3 times with PBS(+), 3D gels were permeabilised for 30 min at room temperature with the permeabilisation solution: PBS(+) with 0.5% Triton X-100 (Sigma) and 5% donkey serum (Millipore). They were incubated with diluted primary antibodies overnight at 4 °C. The cells were washed with PBS(+)/0.5% Triton several times, and then incubated with diluted secondary antibodies for 40 minutes at room temperature. Antibodies were diluted with permeabilisation solution. Then the cells were washed with PBS(+)/0.5% Triton followed by a PBS(+) wash, and their nuclei were stained with 4′,6-diamidino-2phenylindole dihydrochloride (DAPI, Sigma). Primary and secondary antibodies are listed in Supplementary Table S2. Normal goat IgG (Santa Cruz Biotechnology, Santa Cruz, CA), negative control mouse IgG1 (Dako, Glostrup, Denmark), and normal rabbit IgG (Santa Cruz Biotechnology) were used as negative controls for the appropriate antibodies. Cysts were imaged under confocal microscopy LSM700 and analysed by ZEN 2009 Light Edition software (Carl Zeiss, Jena, Germany).

### Flow cytometric analysis

Cultured cells were detached from plate and isolated by trypsin-EDTA treatment followed by filtration with a 50-μm nylon filter. Thereafter, cells were incubated with specific cell surface antibodies, such as APC-conjugated anti-CD133 antibody and PE-conjugated various antibodies, at 4 °C for 1 h. After washing with PBS/3% FBS, cells were analysed and sorted using FACSAria III^TM^ (BD Bioscience).

### Real-time RT-PCR analysis

Total RNA was extracted from primary cells and cyst cells using RNA iso Plus (TAKARA, Shiga, Japan). First-strand cDNA was synthesised using a High Capacity cDNA Reverse Transcription Kit (Life Technologies) or ReverTra Ace qPCR RT Master Mix with genome remover (TOYOBO, Osaka, Japan) and used as a template for PCR amplification. For semi-quantitative RT-PCR, cDNA samples were normalized by the number of hypoxanthine phosphoribosyltransferase 1 (*Hprt1*) mRNA copies. The Universal Probe Library (Roche Diagnostics, Basal, Switzerland) was used for quantitative RT-PCR assays of *Albumin*, *Ck19*, *Ck7*, *Cftr*, *Hnf1β*, *Hnf6*, *Tat*, *Cyp3a11*, *Grhl2*, *Sox9*, *Lgr4*, *Lgr5*, *Lgr6*, *Erbb1*, *Erbb2*, *Erbb3* and *Erbb4* transcripts. The primer sequences and probe numbers for each gene are shown in Supplementary Table S3.

### Analysis of efflux ability

In total, 25 mmol of rhodamine 123 was added to the medium of cysts derived from cultured cells and incubated for 5 min. Some of them were incubated with 100 μM verapamil for 30 min before adding the dye. After washing with PBS 3 times, the 3D culture media were added and incubated for 40 min at 37 °C. Images were acquired with a Floid^TM^ Cell Imaging Station (Life Technologies) and AxioVision Z1 microscopy (Carl Zeiss, Oberkochen, Germany).

### Colony formation assay

E13 LPCs (Dlk1^+^CD133^+^ cells) and neonatal progenitor cells (CD133^+^ cells) from the non-haematopoietic cell fraction were purified using flow cytometry (FACSAria III^TM^). Purified cells were inoculated at a low density (280 cells/cm^2^) onto feeder cells. Mitomycin-C treated mouse embryonic fibroblasts (2 × 10^5^ cells per 1 well of a 12-well culture plate) were used as feeder cells. Cells were cultured with a colony formation medium, as previously described^22^. After 7 to 10 days of culture, cells were fixed with 4% paraformaldehyde and stained with the appropriate antibodies.

### Statistical analysis

Microsoft Excel 2010 (Microsoft, Redmond, WA, USA) was used to calculate standard error (SEM) and a Student’s two-tailed test was used to obtain statistically significant differences between samples.

## Additional Information

**How to cite this article**: Anzai, K. *et al*. Foetal hepatic progenitor cells assume a cholangiocytic cell phenotype during two-dimensional pre-culture. *Sci. Rep.* **6**, 28283; doi: 10.1038/srep28283 (2016).

## Supplementary Material

Supplementary Information
