# Supplementary material for: Foetal hepatic progenitor cells assume a cholangiocytic cell phenotype during two-dimensional pre-culture
Source: Sci Rep. 2016 Jun 23;6:28283. doi: 10.1038/srep28283 (PMC4917868; doi:10.1038/srep28283)
Supplement: Supplementary Information [file srep28283-s1.pdf]

Foetal hepatic progenitor cells assume a cholangiocytic cell phenotype during two-dimensional pre-culture

Kazuya Anzai, Hiromi Chikada, Kota Tsuruya, Kinuyo Ida, Tatehiro Kagawa, Yutaka Inagaki, Tesuya Mine, and Akihide Kamiya

Supplementary Figure Legends

Supplementary Figure 1

Expansion and differentiation of foetal LPCs in 2D pre-culture. Primary cells and cultured cells, which were 2D pre-cultured for 1 day, 3 days or 5 days, were stained with antibodies against a cholangiocytic marker *CK19* or a proliferation marker *Ki-67*. Nuclei were stained with DAPI. Scale bars, 100 μm.

Supplementary Figure 2

Number of cells before and after 7 days pre-culture. Primary E13 LPCs were inoculated onto gelatine-coated culture plates ($1x10^5$ cells per well of a 24-well culture plate or $2x10^5$ primary cells per well of a 12-well culture plate). Cells were cultured for 7 days and the number of cells was counted. Results are presented as mean ± SEM. Statistical analysis was performed with Student's *t*-test. n=40 (24-well plates) and n=9 (12-well plates).

Supplementary Figure 3

Flow cytometric analysis to LPCs pre-cultured with or without cytokines. The expression of PE-tagged antigens was hardly influenced by the addition of cytokines, but the expression of CD133 decrease under the stimuli of OSM or HGF.

Supplementary Figure 4

Expression of cell signalling receptors during *in vitro* cholangiocytic formation. (Upper panel)

Expression of ErbB family genes of primary cells, cultured cells, and cysts derived from cultured cells were analysed by using quantitative RT-PCR. (Lower panel) Expression of the Lgr family genes of primary cells and cysts derived from cultured cells were analysed by using quantitative RT-PCR. Expression level of primary cells was set to 1.0. Mock, cells were pre-cultured without cytokines.

Supplementary Figure 5

Cysts derived from neonatal $CD133^+$ cells expressed *HNF4*α and *Ki-67*. Cysts were stained with anti-*HNF4*α and *Ki-67* antibodies. Arrowheads indicate *Ki-67*-positive cells. Scale bars, 100 μm.

Supplementary Figure 6

Colony formation assay of E13 foetal LPCs and $CD133^+$ neonatal liver cells. Progenitor cells were purified from E13 and neonatal livers. Primary E13 LPCs, E13 LPCs pre-cultured on gelatine-coated dishes, and neonatal progenitor cells were cultured on MEF feeder cells. Resulting colonies were stained with anti-*ALB* and -*CK19* antibodies (left panel). Isotype control (right panel). Red, *ALB*; green, *CK19*. (i) Colonies derived from primary E13 $CD133^+$ LPCs. (ii) Colonies derived from E13 $CD133^+$ pre-cultured LPCs. (iii) Colonies derived from $CD133^+$ neonatal liver cells.

Supplementary Table S1 Cell surface antigens for flow cytometric analysis

	Fluorescent Conjugate	Catalogue Number	Isotype	Company
CD9	PE	124805	Rat IgG2a	BioLegend
CD24	PE	101807	Rat IgG2b	
CD26	PE	137803	Rat IgG2a	
CD29	PE	102207	American Hamster IgG	
CD47	PE	127507	Rat IgG2a	
CD54	PE	116107	Rat IgG2b	
CD63	PE	143903	Rat IgG2a	
CD73	PE	127205	Rat IgG1	
CD81	PE	104905	American Hamster IgG	
CD98	PE	128207	Rat IgG2a	
CD106	PE	105713	Rat IgG2a	
CD121a	PE	113505	American Hamster IgG	
CD147	PE	123707	Rat IgG1	
CD262	PE	119905	American Hamster IgG	
CD276	PE	135605	Rat IgG2a	
CD326	PE	118205	Rat IgG2a	
CD44	FITC	11-0441	Rat IgG2b	eBioscience

Supplementary Table S2 Primary antibodies for immunohistochemistry

Antibody	Company	Catalogue Number	Host Animal	Dilution
ALBUMIN	Betyl laboratory	A90-134A	goat	1:500
CK19	Miyajima et al.	-	rabbit	1:2000
β-CATENIN	BD Pharmingen	610154	mouse	1:1000
aPKC	SantaCruz Biotechnology Inc	sc-216	rabbit	1:250
HNF4α	SantaCruz Biotechnology Inc	sc-6556	goat	1:400
Ki-67	Abcam	15580	rabbit	1:500

Supplementary Table S3 Primer sequences for quantitative PCR analysis

Gene name		Sequence	Probe number
Hprt	Sense	5'-TCCTCCTCAGACCGCTTTT-3'	#95
	Antisense	5'-CCTGGTTCATCATCGCTAATC-3'	
Albumin	Sense	5'-AGTGTTGTGCAGAGGCTGAC-3'	#27
	Antisense	5'-TTCTCCTTCACACCATCAAGC-3'	
Ck19	Sense	5'-TGACCTGGAGATGCAGATTG-3'	#17
	Antisense	5'-CCTCAGGGCAGTAATTTCCTC-3'	
Hnf1β	Sense	5'-ATGGCTCCCCTCACCATC-3'	#55
	Antisense	5'-GGTTGTAGCGCACTCCTGA-3'	
Hnf6	Sense	5'-GGAGTTCCAGCGCATGTC-3'	#64
	Antisense	5'-CGACGTTGGACGTCTGTG-3'	
Tat	Sense	5'-GGAGGAGGTCGCTTCCTATT-3'	#82
	Antisense	5'-GCCACTCGTCAGAATGACATC-3'	
Cyp3a11	Sense	5'-GGGACTCGTAAACATGAACTTTTT-3'	#53
	Antisense	5'-CCATGTCGAATTTCCATAAACC-3'	
Grhl2	Sense	5'-CCACAGAGCATACTGCCAGA-3'	#32
	Antisense	5'-TCTCTTCATCCCGGATTTTTC-3'	
Ck7	Sense	5'-GGAGATGGCCAACCACAG-3'	#41
	Antisense	5'-GGCCTGGAGTGTCTCAAACTT-3'	
Cftr	Sense	5'-CAGCAGCTCAAACAACTGGA-3'	#51
	Antisense	5'-TGTCACAAGGTGGGTGAAAA-3'	
Sox9	Sense	5'-CAGCAAGACTCTGGGCAAG-3'	#66
	Antisense	5'-TCCACGAAGGGTCTCTTCTC-3'	

Gene name		Sequence	Probe number
Lgr4	Sense	5'-CAGTTACCAGAAGATGCATTTAAGAA-3'	#20
	Antisense	5'-CAAGGCTTTTGGGTGGATAA-3'	
Lgr5	Sense	5'-CTTCACTCGGTGCAGTGCT-3'	#60
	Antisense	5'-CAGCCAGCTACCAAATAGGTG-3'	
Lgr6	Sense	5'-AGCTTCAGCCGGGTCTCT-3'	#17
	Antisense	5'-AGAGGTGGTTCCCTGAGAGC-3'	
Erbb1	Sense	5'-GCCACGCCAACTGTACCTAT-3'	#107
	Antisense	5'-GCCACACTTCACATCCTTGA-3'	
Erbb2 (Her2)	Sense	5'-TCAACTGCACCCACTCATGT-3'	#85
	Antisense	5'-CTGGCTCTCTGCTCTGCTG-3'	
Erbb3	Sense	5'-CTGCCACGAGAACTGCAC-3'	#93
	Antisense	5'-TGCTTGGCCTAAACAGTCTTG-3'	
Erbb4	Sense	5'-AATGCTGATGGTGGCAAGA-3'	#21
	Antisense	5'-CATCACTTTGATGTGTGAATTTCC-3'	

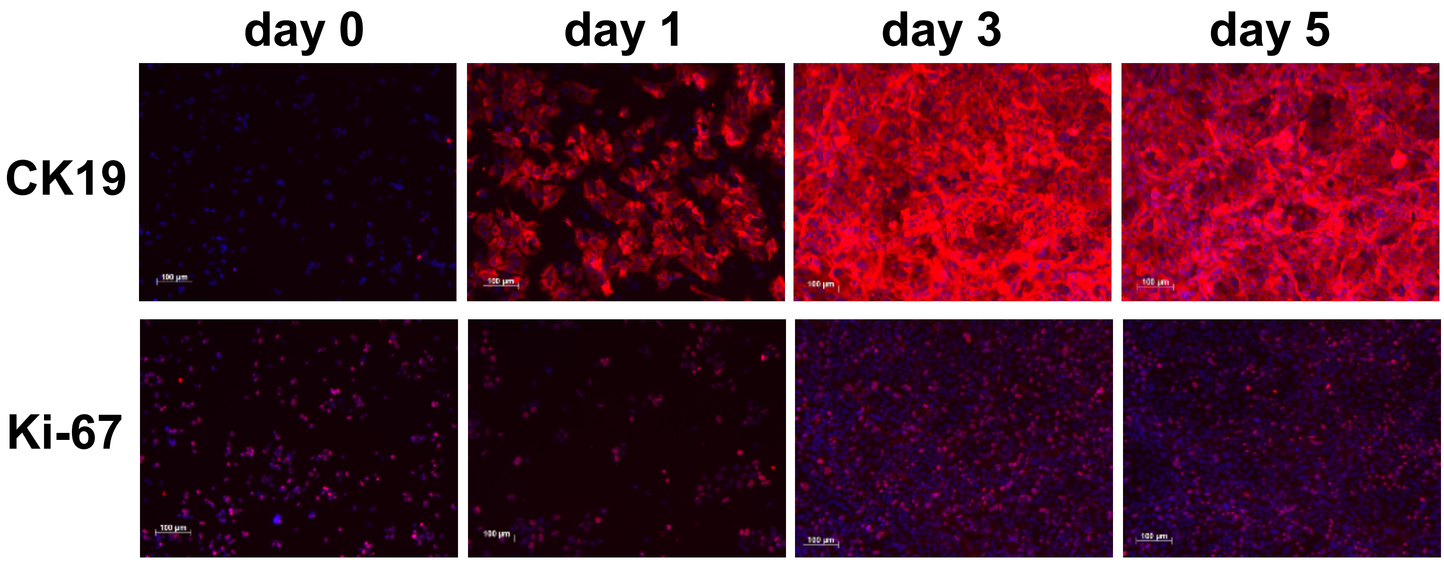

Anzai et al., Supplementary Figure 1

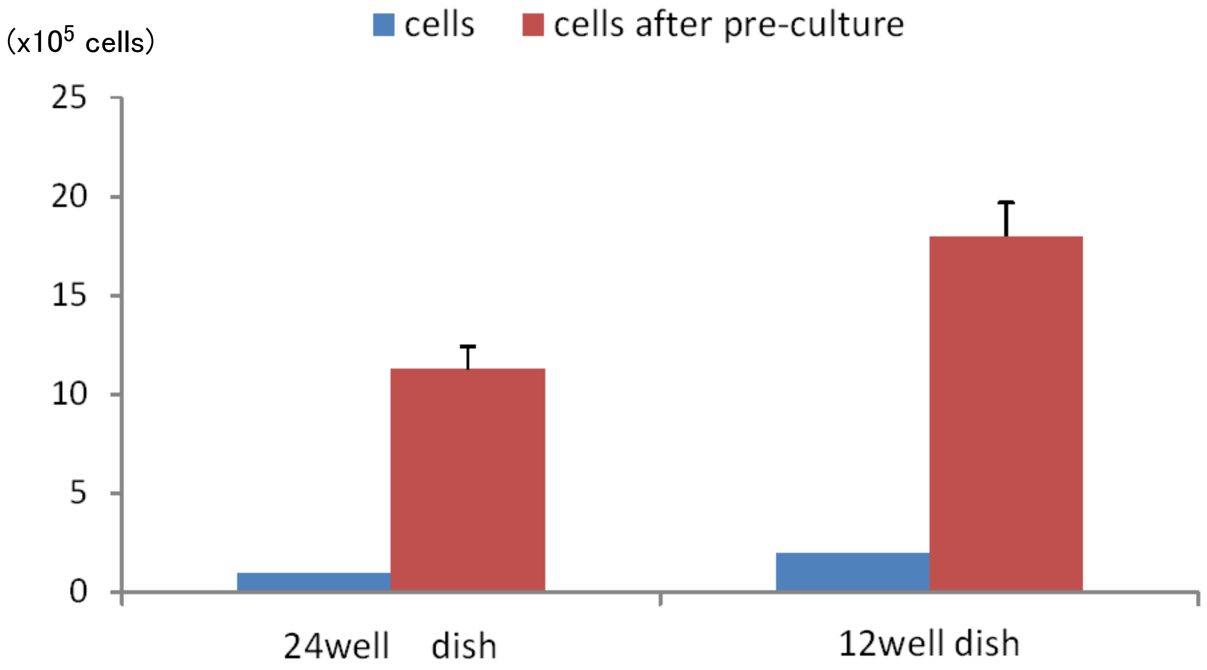

Anzai et al., Supplementary Figure 2

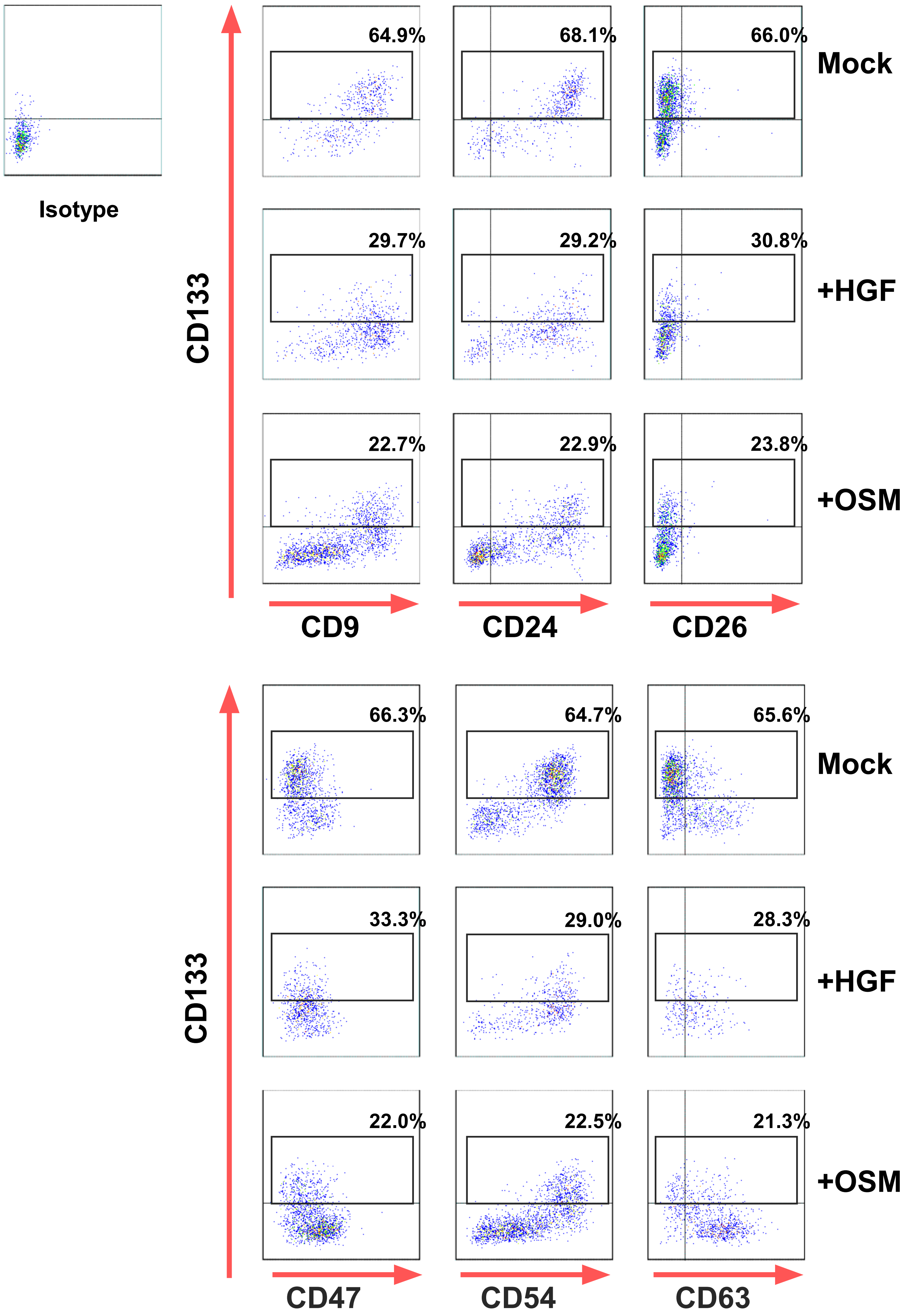

Anzai et al., Supplementary Figure 3

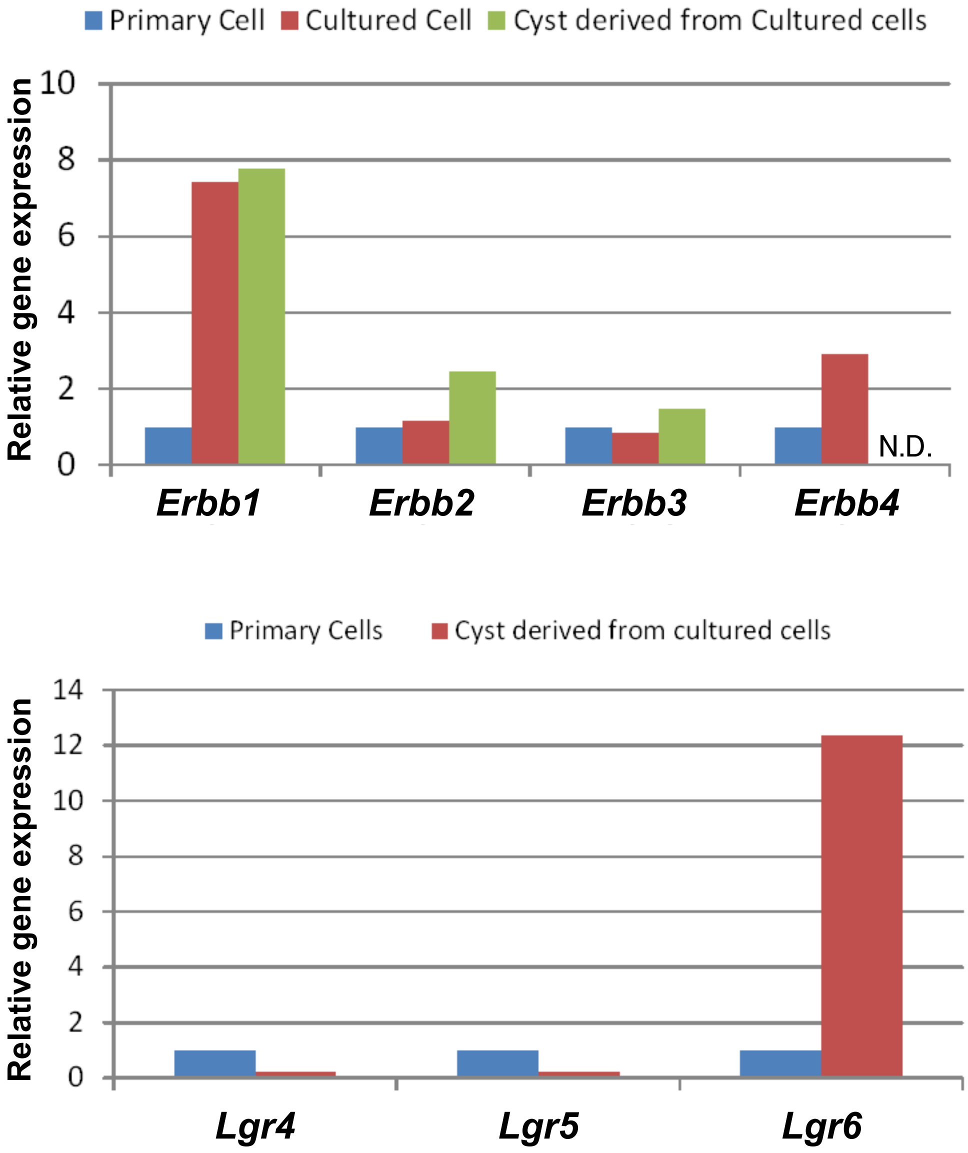

Anzai et al., Supplementary Figure 4

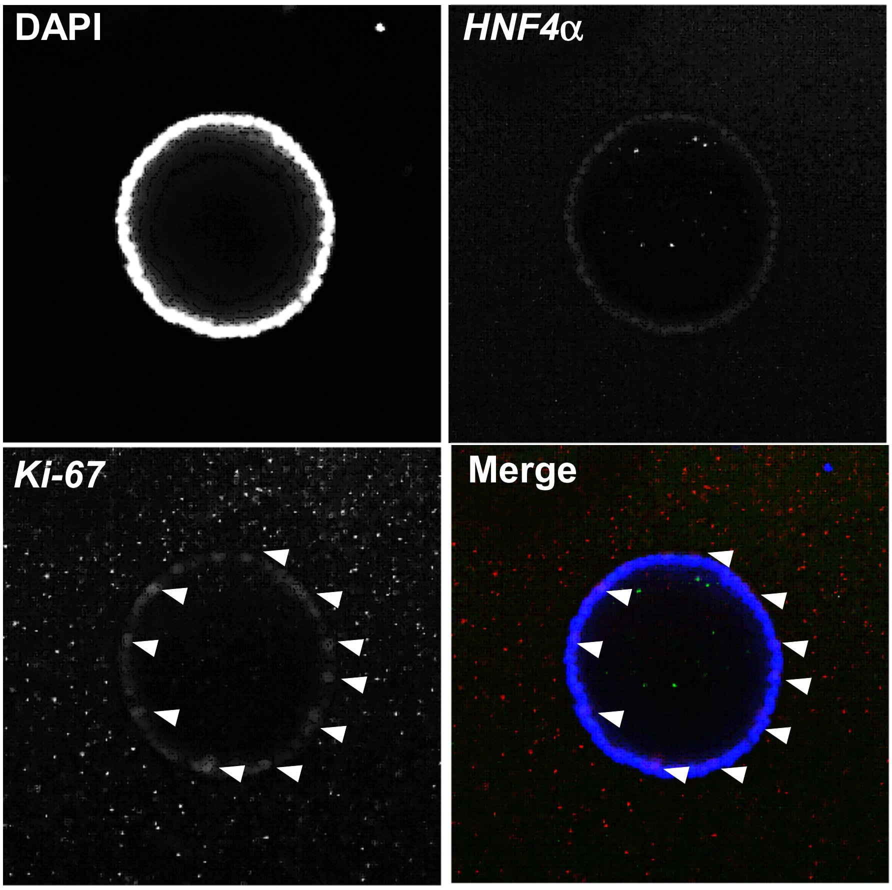

Anzai et al., Supplementary Figure 5

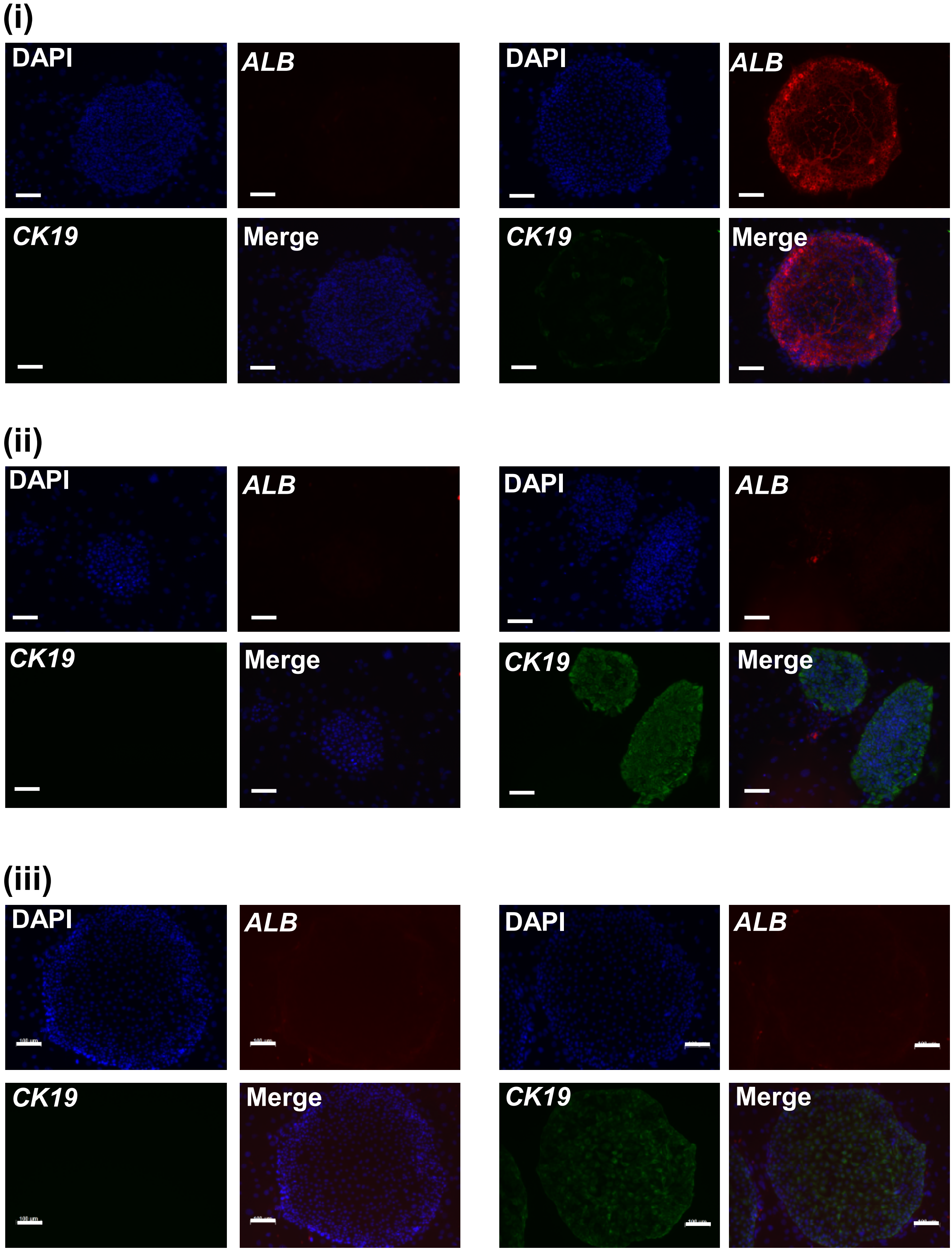

Anzai et al., Supplementary Figure 6
